# Intention to khat chewing among youths in Raya-Azebo district, southern zone of Tigray, Ethiopia: application of the theory of planned behavior

**DOI:** 10.3389/fpubh.2024.1417874

**Published:** 2025-01-07

**Authors:** Abadi Hailay Atsbaha, Adugnaw Berhane Mekonnen, Bezawit Ketema, Tigist Haile Gebrehiwot, Hirut Teame Gebru, Embay Amare Alemseged, Yonas Angaw, Haftay Gebremedhin, Fre Gebremeskel, Hagos Degefa Hidru, Zenawi Hagos Gufue

**Affiliations:** ^1^Department of Public Health, College of Medicine and Health Sciences, Adigrat University, Adigrat, Ethiopia; ^2^Department of Preventive Medicine, School of Public Health, College of Health Sciences, Addis Ababa University, Addis Ababa, Ethiopia

**Keywords:** khat, khat chewing, youths, intention, theory of planned behavior, Raya Azebo, Ethiopia

## Abstract

**Background:**

Khat chewing has become a global phenomenon, resulting in significant physical and mental health issues as well as socioeconomic crises. However, evidence is scarce on Ethiopian youths’ behavioral intentions toward khat chewing, particularly in the Raya-Azebo district of the southern zone of the Tigray region. As a result, this study aimed to assess the intention to chew among youths in Raya-Azebo district, Southern Tigray, Ethiopia.

**Methods:**

A community-based cross-sectional survey was conducted among 627 youths in northern Ethiopia’s Raya-Azebo district. Data were collected using a structured, interviewer-administered questionnaire. A multivariable linear regression model was used to predict the contribution of independent variables and identify variables strongly associated with chewing among youths.

**Results:**

A considerable proportion, 192 (30.62%) of youths, had the intention to chew khat in the next 6 months. The component of the theory of planned behavior independently explained the variance in intention to chew by 83%. The strongest predictors of intention to chew were attitude (*β* = 0.35, *p* < 0.001), subjective norm (*β* = 0.297, *p* < 0.001), and perceived behavioral control (*β* = 0.15, *p* = 0.01).

**Conclusion:**

Behavioral intention toward khat chewing was a function of attitude, subjective norm, and perceived behavioral control toward khat chewing. Strategies to empower youths to change a positive attitude toward khat chewing, programs targeted at resisting social pressures, and increasing self-efficacy to combat chewing are needed.

## Background

Khat (*Catha edulis Forsk*) is an evergreen flowering tree found in Yemen by botanist Forskal in 1762. The plant belongs to the *Spinosa genus*; however, currently, it is classified under the family *Celastraceae*. Khat grows well and is primarily cultivated at high altitudes in East Africa and the Arabian Peninsula (1). Khat produces cathinone and cathine which are accountable for physical and mental health problems (2). Simultaneous intake of khat with drugs can lead to interaction and reaction (3). Ethiopia is the origin of khat, and chewing began in the 15th century, long before coffee was introduced. Ethiopia continues to produce the world’s largest khat (1, 4). The exact global prevalence of khat chewing is unknown, but it is estimated that between 5 and 10 million people engage in this practice. It is widely chewed in southwestern Arabia and eastern Africa (5, 6).

Khat consumption is widespread in Yemeni society, where it is commonly consumed at social events (7). In Ethiopia, khat chewing is becoming a habit and spreading to new areas where it is not cultivated. In Ethiopia, young people, including high school, college, and university students, are more likely to use khat (8). According to the 2016 Ethiopian Demographic Health Survey (EDHS), 12% of women and 27% of men reported having used khat. The highest prevalence of khat use was among men (34%) and women (15%) aged 30–34 (9). The burden of lifetime khat consumption rests in Yemen (43.27%), Saudi Arabia (37.32%), and Ethiopia (24.82%) (2).

Ethiopia signed and ratified various International Conventions on Drug Control (ICDC) at different times. The Convention against the Illicit Traffic of Narcotic Drugs and Psychotropic Substances was signed in 1971 and 1988. In 1993, the government formulated a detailed policy to control and prevent the production, trafficking, and use of narcotic drugs and psychotropic substances (10, 11). Despite this, there has been an increase in substance abuse, khat production, and chewing across the country, as well as a decline in cross-border drug trafficking. There are several reasons why the rules and regulations have failed to achieve the desired results.

First, there has been a lack of community participation. Furthermore, khat chewing is culturally acceptable, making it difficult to impose regulations, and countries do not agree on how to control substances. The failure can also be attributed to a lack of a multi-agency approach and a comprehensive community education campaign. Finally, there is a conflict between the human right to health and the human right to adequate food, making it difficult to achieve a balance (10, 11).

Different theories and models are applied to change and predict behaviors, particularly in substance use, such as cigarette smoking, alcohol drinking, and khat chewing. The transtheoretical model (TTM) is applied to change unwanted problematic behavior to desired behavior through stages and a process of change (12). TTM is used to assess individuals’ stages of change from actual undesired behavior to desired behavior; i.e., from smokers, and khat chewers to nonsmokers (13, 14) and nonchewers (15, 16) respectively. Social learning theory (SLT) is another theory used in substance use in both acceptable and deviant actual behaviors. It assumes that behavior is learned and learning occurs through differential association, reinforcement, definition, and imitation and is influenced by attention, attitude, motivation, and emotions. Engagement, replacement, or elimination of substance use can be more or less problematic according to the social influences and the extent to which individuals are reinforced for the behavior (17–21).

The theory of planned behavior (TPB) is the extension of the Theory of reasoned action (TRA) and is used to predict an individual’s intention to engage in new behavior at a specific time and place. Behavioral intention is the most immediate and best predictor of behavior which in turn is determined by three conceptually independent determinants postulated by TPB. The first is the attitude toward the behavior, the second predictor is a social factor termed subjective norm, and the third antecedent of intention is the degree of perceived behavioral control (PBC). The relative importance of attitude, subjective norm, and perceived behavioral control in the prediction of intention is expected to vary across behaviors and situations. Therefore, attitude may have a significant impact on intention or attitude, and subjective may have a significant impact on intention or other possible (22, 23).

TPB has three types of belief and it explains their difference among them. Behavioral beliefs are assumed to influence attitudes toward the behavior, normative beliefs that constitute the underlying determinants of subjective norms, and control beliefs that provide the basis for perceptions of behavioral control (23). TPB constitutes a proficient framework and Ajzen noticed the possibility of adding other determinants only if they contribute to the variance explained in behavioral intention (24, 25). In this particular study, a conceptual framework was adapted from TPB and modified using additional elements with TPB to maximize the variance explained in the outcome variable. Socio-demographic characteristics, knowledge, and previous experiences as additional predictors of behavioral intention based on the reading of different research works of literature.

Previous researches focused on the prevalence, causes, and consequences of khat chewing but did not examine attitudes, subjective norms, perceived behavioral control, or behavioral intention toward khat chewing. The theory of planned behavior (TPB) looks into the relationship between attitudes, social norms, perceived behavioral control, and the intention to chew khat. However, the majority of the research has been carried out in institutions that may not be representative of the wider community. The purpose of this study was to assess the likelihood of khat chewing in a community over the next 6 months using a representative sample from the Raya-Azebo district. This study’s findings will provide valuable insights to policymakers, health professionals working on substance abuse, health facility experts and managers, as well as researchers interested in this area.

## Methods

### Study design and settings

A community-based cross-sectional study was conducted from March to May 2019 among 627 randomly selected youths from Raya Azebo district in southern Tigray, Ethiopia. Raya-Azebo is situated in the southern part of the Tigray regional state, 678 kilometers north of Addis Ababa. According to Central Statistical Agency (CSA) data from 2007, the district’s total population is 135,870.

### Study participants

The study included non-khat users whose ages ranged from 15 to 24 years. To reduce deviant perceptions of chewing behavior caused by increased awareness, information, and exposure, people who had lived in the study area for less than 6 months were excluded.

### Sample size determination and sampling technique

The sample size was calculated using a single population formula for the mean difference of a finite population. To predict intention, the variance was set to 50% (*σ* = 0.5) with a 95% confidence interval (*α* = 0.05) and a 5% tolerance for marginal sampling error. With these assumptions, the sample size was 384; adding a 10% non-response rate and accounting for the 1.5 design effect resulted in a final sample size of 641 youths. Adjusting the sample size for the non-response rate is crucial to keep our sample size representative. The final sample size was determined by adding a non-response rate to the calculated sample by using the loss adjustment formula. The adjustment was made by dividing the calculated sample size (n) by (1-X) where “X” is the proportion of the expected to withdraw or non-response rate. *N* = *n*/(1-X), *N* = 384/ (1–0.1) = 384/0.9 = 426.67 ~ 427. Then, 427*1.5 = 640.5 ~ 641.

Multistage cluster sampling was used to reach the household level. The final sample size was proportionally allocated based on the households of each kebele, and then a simple random sampling technique was applied to select study participants.

### Data collection tool and procedure

A context-specific, pretested, and structured interviewer-administered questionnaire was used to collect the needed data from the participants. The questionnaire was developed based on TPB questionnaire development guidelines (26), different related kinds of literature, and after conducting an elicitation study in the study area to elicit the commonly held salient beliefs, indirect measures of attitude, subjective norm, and PBC. Language experts translated the questionnaire into Tigrigna and then back into English to maintain the consistency of the tool. Pretesting was carried out in a different district on 5% of the total sample size 1 week before data collection began. Trained data collectors and supervisors were in charge of data collection and supervision, respectively.

### Measurements and scoring

The measurement and scoring methods applied in this study were based on the TPB construct questionnaire development guideline (26). The intention was measured by four items with seven Likert scales ranging from strongly disagree to strongly agree. It was calculated by adding the scores of the items, and the total score of the items ranges from 4 to 28. If the calculated intention composite score is a high composite score; they have an intention toward khat chewing. If the calculated intention composite score is a low composite score, they have no intention toward khat chewing.

The direct attitude was measured by four items with seven Likert scales on the Semantic Differential Scale (SDS), ranging from extremely bad to extremely good. It was calculated by adding the scores of the items, and the total score of the items ranges from 4 to 28. If the calculated attitude composite score is a high composite score, they have a positive attitude toward khat chewing. If the calculated attitude composite score is a low composite score, they have a negative attitude toward khat chewing. Behavioral belief was measured using six items with seven Likert scales on bipolar differential scales. Six items with seven Likert scales were used to measure the evaluation of khat chewing beliefs.

A new variable was formed that represents the weighted score by multiplying each behavioral belief with an evaluation of the belief. Finally, the summation of each product of beliefs was done to create an indirect attitude as a new variable. One behavioral belief measurement item is followed by a single measurement item of behavioral outcome evaluation. Hence, indirect attitude was measured using 12 items with seven Likert scale and the total score ranges from (−126) to (+126). Besides, the normative belief of khat chewing with motivation to comply and each control belief with perceived power were weighted to create new variables that represent weighted scores for each normative belief and control belief, respectively.

The summation of each weighted belief was done to create a new indirect subjective norm and indirect PBC. Indirect subjective norm was measured by 8 items with seven Likert scale (4 items of normative belief and 4 items of motivational to comply) and the total score ranges from (−84) to (+84). Each question items of motivational to comply is placed next to a single question item of normative belief. Besides, the indirect PBC was measured by 18 items with seven Likert scale (9 items of control belief and 9 items of perceived power) and the possible total score ranges from (−189) to (+189). Each questioning item of perceived power is placed next to a single question item of control belief.

Regarding subjective norm, it was measured by four items with seven Likert scales ranging from strongly disagree to strongly agree or should to should not. It was calculated by adding the scores of the items, and the total score of the items ranges from 4 to 28. If the calculated subjective norm composite score is a high composite score, the social pressure is in favor of khat chewing. If the calculated subjective norm composite score is a low composite score, the social pressure is against khat chewing.

Perceived behavioral control was measured by four items with seven Likert scales on SDS ranging from strongly disagree to strongly agree or extremely difficult to extremely easy. It was calculated by adding the scores of the items, and the total score of the items ranges from 4 to 28. If the calculated perceived behavioral control composite score is high, they have a strong perceived ability, less difficulty, or are easy to chew. If the calculated perceived behavioral control composite score is a low composite score, they have a weak perceived ability or difficulty chewing khat.

### Data analysis

Data were checked for clarity, completeness, and consistency, edited, coded, and entered into Epi Data version 4.4.2, and then exported to SPSS version 25 for analysis. Descriptive statistics were presented in mean (standard deviation) or median (inter-quartile range, IQR) for numerical variables, depending on data distribution and frequency percentage for the categorical variables. An independent *t*-test and one-way ANOVA with post-hoc comparisons were done to see the association and mean difference between intention and categorical independent variables. Bivariate correlation analysis was done between the indirect and direct measures of the same construct (direct attitude versus indirect attitude, direct subjective norm versus indirect subjective norm, and direct PBC versus indirect PBC) to check the validity of the measurement tool.

Next, to bivariate analysis and selection of variables with a *p*-value <0.25, a multiple linear regression was done to see the contribution of direct TPB constructs on behavioral intention to chew khat, show the standardized regression coefficient (β), and control confounders. The summary measures of the estimated unstandardized and standardized regression coefficient (*β*) with a 95% confidence interval were presented, a *p*-value <0.05 was used to declare statistical significance, and the goodness of fit of the model was assessed using R-square and adjusted R-square (Adj. R^2^).

## Results

The study was conducted among 627 randomly selected youths of the Raya-Azebo district, with a response rate of 97.82%. The median age of the participants was 20, with an IQR of 18–23 years. Out of the study participants, 418 (66.7%) were males, 284 (45.30%) were Orthodox, 343 (54.70%) were Muslims, 410 (65.39%) were single, and 162 (25.84%) were married. All the interviewed youths were Tigrayans (Table 1).

**Table 1:** Socio-demographic characteristics of study participants in Raya-Azebo district, Southern Tigray, Ethiopia, 2019 (*n* = 627).

Variables	Characteristics	Frequency (*n*)	Percentage (%)
Age (years)	15–19	232	37.00
	20–24	395	63.00
Sex	Male	418	66.67
	Female	209	33.33
Marital status	Single	410	65.39
	Married	162	25.84
	Divorced	55	8.80
Religion	Orthodox	284	45.30
	Muslim	343	54.70
Education	Unable to read and write	91	14.51
	Able to read and write but no formal education	160	25.52
	Grade 5–8	116	18.50
	Grade 9–10	181	28.87
	Grade 11–12	35	5.58
	Diploma	22	3.5
	Others	22	3.5
Occupational status	Farmer	197	31.42
	Spouse	56	8.93
	Private employee	29	4.63
	Student	194	30.94
	Daily laborer	11	1.75
	Government employee	30	4.78
	Others	110	17.54
Income (Ethiopian Birr)	≤ 100	19	3.0
	101–299	64	10.2
	300–499	19	3.0
	500–999	108	17.2
	≥ 1,000	231	36.8
Source of income	Agriculture	235	37.5
	Parents	181	28.9
	Salary	58	9.3
	Khat selling	51	8.1
	Coffee/tea and shopping	39	6.2
	No source of income	35	5.6
	Others	28	4.5

### Knowledge of khat and past experiences of substance abuse

All participants had ever heard about khat chewing, and the information was found from teachers, health professionals, religious leaders, administrative leaders, friends, and social media. Regarding knowledge, 500 (79.75%) participants agreed that chewing causes addiction and diseases. Among these, 93 (18.6%), 219 (48.8%), and 188 (37.6%) relied on the fact that chewing causes physical health problems, mental health problems, and both, respectively. According to the previous behavioral experiences, 263 (41.95%), 261 (41.63%), and 202 (32.22%) participants had experienced khat chewing, other substances, and both, respectively. Among other substance users, 80 (31.01%) were cigarette smokers, 110 (38.7%) were shisha smokers, and 130 (50.39%) were alcohol drinkers.

### Analyzing the mean difference between predictor variables with intention

#### Socio-demographic variables and intention to khat chewing

An independent *T*-test was conducted to determine if there is a difference existed between the mean of the two groups of independent variables to the intention of khat chewing. In addition to this One-way ANOVA was done to check the existence of and calculate the difference among the mean of the three and above groups of independent variables to the intention of khat chewing. From the independent *T*-test analysis done, there was a statistically significant difference between Orthodox followers and Muslims mean in the intention scores of khat chewing (*t* (625) = −10.898, *p* < 0.01, 95% CI = −8.52682, −5.92304). The mean values indicate that Muslims had more intention toward khat chewing (*n* = 343, *M* = 15.8834) than orthodox followers (*n* = 284, *M* = 8.6585) (Table 2).

**Table 2:** Summary of Independent t-test of socio-demographic characters with intention among the youth of Raya-Azebo, Southern Tigray, Ethiopia, 2019.

Variables	Values	*N*	Mean	SD	*T*	*p*-value	95% CI		Effect size
							Lower	Upper	
Sex	Male	418	13.06	9.11	1.778	0.041	−0.142	2.85	0.005
	Female	209	11.71	8.75					
Religion	Orthodox	284	8.66	6.27	−10.898	<0.001	−8.53	−5.92	0.160
	Muslim	343	15.88	9.61					
Age (year)	15–19	232	10.595	8.69	−4.356	<0.001	−4.643	−1.757	0.029
	20–24	395	13.795	8.99					

SD, standard deviation; *T*, *T*-test.

One-way ANOVA was done for the socio-demographic characters that have more than two levels of groups. The results of the analysis indicate that the marital status of participants had a significant effect on the intention toward khat chewing (*F* (3, 623) = 8.506, mean square = 666.10, *p* < 0.001). The mean values indicate that divorced had more intention toward khat chewing (*n* = 53, *M* = 18.39) than single (*n* = 410, *M* = 12.09) and married (*n* = 162, *M* = 11.97). A *post hoc* comparison was used to show the location of the difference between marital status. The results of post hoc comparison analysis indicate that the divorced marital status was significantly different from both the single and married marital status. The results show that the overall difference in intention of khat chewing among the marital status was because of the significantly greater amount of intention toward khat chewing by the participants in the divorced marital status (Table 3).

**Table 3:** Summary of one-way ANOVA for socio-demographic variables with intention among the youth of Raya-Azebo, Southern Tigray, Ethiopia, 2019.

Variables	M - square	*F*	*p*-value	Effect size
Marital status	666.10	8.506	<0.001	0.039
Educational level	899.64	12.52	<0.001	0.124
Occupation	1238.77	17.72	<0.001	0.146
Source of income	1464.21	21.70	<0.001	0.174
Income	1033.51	13.84	<0.001	0.109

#### Reliability and correlation of theory of planned behavior constructs

**Figure 1:**
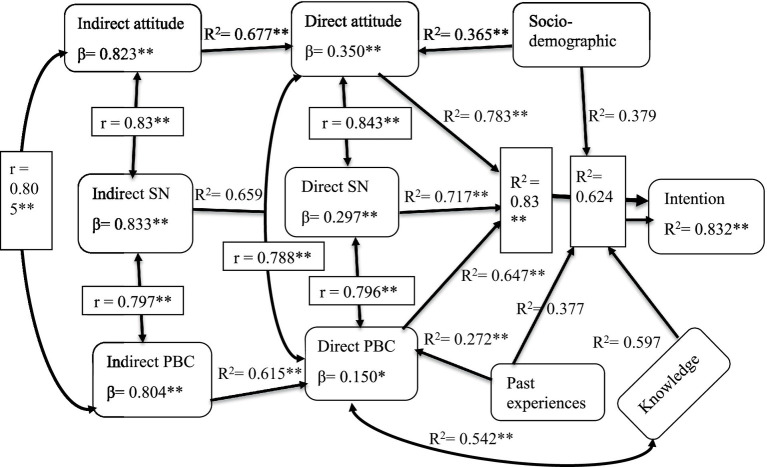
Summary of correlation and regression result of predictive model TPB, socio-demographic, knowledge and past behavioral experiences to predict of intention to khat chewing among youths of Raya-Azebo district, Southern Tigray, Ethiopia, 2019. This figure shows the path of how the variety of external independent variables and indirect and direct components of theory planned behavior predicted the intention to khat chewing among youths. ** = statistically significant at *p* < 0.001; * = statistically significant at *p* < 0.05; r, correlation between variables; *R*^2^ (R-squared), coefficient of determination; *β*, standardized regression coefficient; SN, Subjective Norm; PBC, Perceived Behavioral Control.

Before the use of the instrument, the test–retest reliability was done to determine the reliability of the indirect measure of the TBP model (Figure 1). The correlation coefficient for the TPB constructs is between 0.96 and 0.976, indicating that the measurement of TPB is valid. Internal consistency among the items of the TPB model constructs Cronbach *α* coefficient was between 0.704 and 0.977, indicating that the internal consistency reliability of all items is good (Table 4).

**Table 4:** Descriptive statistics theory of planned behavior variables’ mean score, internal consistency, and test–retest reliability, among youths of Raya-Azebo district, Southern Tigray, Ethiopia, 2019 (*n* = 627).

Variables	Items	Possible min-max	Observed min-max	Mean	SD	α & r
Intention	4	4–28	4–28	12.611	9.007	0.977^¶^
Direct Attitude	4	4–28	4–28	11.145	8.856	0.958^¶^
Direct SN	4	4–28	4–28	11.963	7.704	0.829^¶^
Direct PBC	4	4–28	4–28	14.537	6.984	0.704^¶^
Indirect attitude	12	−126-(+126)	−126-(+126)	−18.423	45.861	0.976^¶¶^
Indirect SN	8	−84-(+84)	−84-(+84)	−26.362	52.334	0.970^¶¶^
Indirect PBC	18	−189-(+189)	−140-(+163)	−10.499	71.462	0.960^¶¶^

SD, standard deviation; ^¶^ = α = Cronbach’s alpha for the direct measures’ internal consistency, ^¶¶^ = r = average item-total correlation for the indirect measures’ temporal stability, SN, subjective norm; PBC, Perceived behavioral control; Min-max, minimum and maximum values.

The correlation between the direct and respective indirect measures of TPB was 0.784 to 0.823, strongly positive (*r* > 0.7), which indicates that the indirect measures are valid, well-constructed, and adequately cover the breadth of measured constructs, which means the direct measure is also reliable and valid (Table 5).

**Table 5:** Correlations (Karl Pearson’s “r”) of indirect and direct measures of theory of planned behavior constructs among youths of Raya-Azebo, Southern Tigray, Ethiopia, 2019 (*n* = 627).

Variables	Attitude	SN	PBC	Indirect attitude	Indirect SN	Indirect PBC
Attitude	1					
SN	0.843**	1				
PBC	0.788**	0.796**	1			
Indirect attitude	0.823**	0.773**	0.725**	1		
Indirect SN	0.833**	0.812**	0.711**	0.830**	1	
Indirect PBC	0.804**	0.758**	0.784**	0.805**	0.797	1

** = Correlation is significant at *p* < 0.01 level (2-tailed), SN, Subjective norm; PBC, Perceived behavioral control.

#### Direct theory of planned behavior components with intention toward khat chewing

All direct components of TPB and intention to khat chewing had a low mean score. Direct PBC, SN, and attitude had a mean score of 14.537 (SD = 6.984), 11.963 (SD = 7.704), and 11.145 (SD = 8.856), respectively (Table 4). The bivariate analysis done indicates that attitude (ß = 0.9, 95% CI 0.863, 0.937), subjective norm (ß = 0.99, 95% CI 0.941, 1.039), and PBC (ß = 1.038, 95% CI 0.977, 1.098) were highly statistically significant in the predicting of intention of youths toward khat chewing and 78.3% of the variation in the prediction of intention toward khat chewing was explained by attitude (R^2^ = 0.783). Subjective norm accounted for 71.7% of the variability in the prediction of intention to khat chewing and 64.7% of the variation in predicting intention to khat chewing was explained by PBC (Table 6).

**Table 6:** Bivariate analysis of theory of planned behavior’s explanatory variables in predicting intention toward khat chewing among youths of Raya-Azebo district, Southern Tigray, Ethiopia, 2019.

Variables	ß	*p*-value	95% CI		R^2^ (R-square)	Adj. R^2^
			Lower	upper		
Attitude	0.900	<0.001	0.863	0.937	0.783	0.782
Subjective norm	0.990	<0.001	0.941	1.039	0.717	0.717
Perceived behavioral control	1.038	<0.001	0.977	1.098	0.647	0.647

Adj. R^2^, Adjusted R-square; ß, Unstandardized regression coefficient.

#### Indirect components of the theory of planned behavior

Indirect attitude was statistically significant in predicting of direct attitude (ß = 0.159, 95% CI 0.15, 0.167). An indirect subjective norm was a predictor of a direct subjective norm (ß = 0.119, 95% CI 0.113, 0.126). Indirect PBC also predicted direct PBC (ß = 0.077, 95% CI 0.072, 0.081). Therefore, the predictive power of the constructs to intention, capturing the dimension of the population interest salient beliefs and validity of the data collection tool was high (Table 7).

**Table 7:** Bivariate analysis of indirect theory of planned behavior’s explanatory variables in predicting of their corresponding direct measure toward khat chewing among youths of Raya-Azebo district, Southern Tigray, Ethiopia, 2019 (*n* = 627).

Variables	ß	*p*-value	95% CI		R^2^ (R-square)	Adj. R^2^
			Lower	upper		
Indirect attitude	0.159	<0.001	0.150	0.167	0.677	0.676
Indirect subjective norm	0.119	<0.001	0.113	0.126	0.659	0.658
Indirect perceived behavioral control	0.077	<0.001	0.072	0.081	0.615	0.615

Adj. R^2^, Adjusted R-square; ß, Unstandardized regression coefficient.

#### Multiple linear regression analysis

In the final regression model, 83.2% of the variability of intention to khat chewing was explained by the independent variables (R^2^ = 0.832, Adj. R^2^ = 0.787, *p* < 0.001). Socio-demographic, knowledge, and past behavioral experience variables explained the model by 62.4%. When the TPB direct measures were added to the final model the variance of the dependent variable was changed by 20.8%. However, the components of TPB independently accounted for 83% of the variability of intention to chew khat which direct measures of TPB exclusively explained it. A unit positive change attitude toward advantage associated with the chewing of khat will change the intention to chew khat by 0.35 while keeping other variables constant (*β* = 0.35, 95% CI 0.196, 0.49).

At the same time, for a unit positive change in the individual’s perception of very important persons thought them to chew khat as a normative action will change the intention to chew khat by 0.297 when the other factors remained unvaried (*β* = 0.297, 95% CI 0.166, 0.490). In addition to this, a unit positive change in the perceived control about facilitators associated with the chewing of khat as a perceived action will change the intention to chew khat by 0.15 by keeping the other variables unchanged (*β* = 0.15, 95% CI 0.055, 0.404) (Table 8).

**Table 8:** Multivariable regression analysis of intention toward khat chewing as a dependent variable predicted by independent variables among youths of Raya-Azebo district, Southern Tigray, Ethiopia, 2019 (*n* = 627).

Variables	ß	*β*	*p*-value	95% CI	
				Lower	Upper
Attitude	0.343	0.350	<0.001	0.196	0.490
Subjective norm	0.328	0.297	<0.001	0.166	0.490
Perceived behavioral control	0.229	0.150	0.010	0.055	0.404

ß, Unstandardized regression coefficient; *β*, Standardized regression coefficient.

R^2^ = 0.832, Adjusted R^2^ = 0.787, F change = 18.591, *p* = <0.001.

## Discussion

This study shows that there was a low intention not to chew khat. However, there were significant numbers that had the intention of chewing. Even though behavioral intention is the most proximal and predominant construct of a behavior, an individual who has a high intention does not mean surely performing that behavior or action. There was no significant association between the socio-demographic characteristics of the participants and their intention to chew. This finding is in line with the study conducted at Jimma University, which found that age, marital status, and family sources of income were not significantly associated with khat chewing (27).

This implies that the prediction of attitude, subjective norm, and perceived behavioral control is not different among the various categories of socio-demographic characteristics of participants. However, the present finding is in contrast with the studies done in Mana district, Nekemete town, and the systematic and meta-analysis was done among Ethiopian University students; sex, religion, marital status, and educational status were significantly associated and predictors of khat chewing (1, 4, 5). The difference could be due to the studies being done in different settings and socio-demographic characteristics age, educational status, and marital status so different among the studies’ participants.

Intention to khat chewing was mainly due to attitude, subjective norm, and perceived behavioral control while the other external to TPB variables were insignificantly predictors. The simultaneous predictive power of attitude, subjective norm, and perceived behavioral control in terms of adjusted R-squared was 82.9%. The theory of planned behavior components and external to TPB variables on intention in terms of R square and adjusted R squared were 0.832 and 0.787, respectively. This finding is near to the perfect relationship and cause-effect level of determination.

This predictive power is higher than the other different systematic reviews, and a meta-analysis was done using TPB to predict the behavioral intention of smoking and alcohol consumption (28, 29). The implication is that the internal consistency among items is higher, and the correlation between the direct measure of the theory of planned behavior and the intention to chew is stronger.

The behavioral intention of khat chewing was primarily under the attitudinal influence. This implies that youths’ favorable attitudes toward khat chewing will lead them to chew khat. In a previous study conducted on the prediction of cigarette smoking, attitude was the strongest predictor of intention to smoke. In a systematic review and meta-analysis done using the theory of planned behavior in predicting alcohol consumption, the attitude was more highly correlated with behavioral intention than the subjective norm, and the subjective norm was also more highly correlated with behavioral intention than perceived behavioral control (28, 30).

However, other previous studies show that attitude was the second and third predictor of behavioral intention to cigarette smoking and the second predictor of intention to alcohol drinking (29, 31, 32). The possible reason for this difference might be the variation in behavior, the population in which the study generalizes results, situations, and circumstances under which the behavior is occurring, according to the theory of planned behavior perspective (24, 33). The implication is an attitude toward a specific behavior might be different in various settings, times, cultures, contexts, and societies based on the knowledge and feeling of specific behavior that occurred or formed in the various areas with different cultural, contextual, and societal make-ups and the changes over times or temporal stabilities of the attitude toward behavior.

As the multivariable analysis depicts, the standardized regression coefficient of the subjective norm was secondarily predicting the intention to chew. This indicates that youths’ social pressures, family, peers, and significant others’ pressure toward khat chewing have a great role in leading them to chew it. Previous studies conducted in Ethiopian settings focused on factors initiating khat chewing: peer pressure, social and psychological reasons, socialization issues, khat is considered a social and cultural construct of community, and having family members and friends who chew khat (1, 4, 5, 27, 34–36). This implies that individuals who matter and approve of their important persons’ approval to them and socio-cultural conditions are influenced to perform the behavior. The implication is having direct relationships with referents who engage in a certain form of conduct, exposure to different sets of values and norms, balancing of expected and actual rewards and punishment, and the definition or judgment to behavior determine the intention and occurrence of the behavior.

In line with the study conducted and meta-analysis done on the prediction of cigarette smoking using the theory of planned behavior, the subjective norm was the second strongest predictor of intention to cigarette smoking (29, 30) and systematic review and meta-analysis done on the theory of planned behavior in predicting alcohol consumption, subjective norm was the second highly correlated to behavioral intention to alcohol drinking (28). In contrast to this, another study conducted to predict intention to alcohol consumption reveals that subjective norm was the strongest predictor of behavioral intention to alcohol consumption (31). This implies that behavior is attributable to the target population, type of actions, contexts, time, and other circumstances in which it occurs.

In line with the systematic review and meta-analysis done on predicting alcohol consumption using the TPB model (28), the present study depicts that perceived behavioral control was the third important and statistically significant predictor of intention to khat chewing. This indicates that youths with high confidence to chew khat are high with an intention to khat chewing. However, a previous study and meta-analysis did show that perceived behavioral control was the primary predictor of intention to cigarette smoking (29, 32) and in opposition to this, perceived behavioral control was an insignificant predictor of intention to alcohol drinking (31). It implies that predictors could be similar and vary in different behaviors, circumstances, contexts, and population groups. The perceived likelihood of constraints and facilitators and the belief ability to start a new behavior determines behavior occurrence and variation could occur due to situations of the impeders and opportunities with their effect on behavior, and the variation settings, cultures, contexts, and the exposure to the behavior.

On regression analysis, indirect components of theory-planned behavior explained the variation of their respective direct components of theory-planned behavior. Indirect attitude, subjective norm, and perceived behavioral control toward khat chewing accounted for 67.7, 65.9, and 61.5% variation respective direct components of theory planned behavior attitude, subjective norm, and perceived behavioral control of khat chewing. This predictive power of indirect components to their respective direct component of theory planned behavior is higher than previous studies conducted using TPB supported by elicitation study (37, 38). The possible explanation related to this might be that there was a variation in the temporal stability of salient beliefs commonly held in the community and a correlation between indirect and corresponding direct measures of the theory of planned behavior.

Regarding the present study, the finding demonstrates the fact that all indirect measures of the theory of planned behavior had significant positive indirect influences on the intention to khat chewing through their corresponding direct measures theory of planned behavior. This finding is supported by the suggestion of the theory of planned behavior principles (24, 33). This finding implies that the commonly held beliefs about the behavior of khat chewing were well explored. Other possible reasons could be a strong correlation between indirect and respective direct components of the theory of planned behavior and the direct component of the theory of planned behavior with the intention to khat chewing as well.

### Strengths and limitations

#### Strengths

The researchers did an elicitation study to explore salient beliefs to design a culturally appropriate survey instrument to measure TPB constructs. These salient beliefs are not observable characters and difficult to find the belief with other methods. The study included different variables except for the constructs of the theory of planned behavior. This made the study more comprehensive than the theory’s constructs. The researchers also did cross-validation the final predictive model using the Stein’s formula and data splitting at random.

#### Limitations

Even though the study used the interviewer-administered questionnaire, it has the potential to introduce social desirability bias. In this study, face validity was done to check the validity of the data collection instrument which is affected by individual subjectivity. The relation between constructs of TPB was measured at the spot not in prospective time intervals.

## Conclusion

This study revealed that a considerable proportion of youths had an intention to chew khat in the next 6 months. Behavioral intention to khat chewing was a function of attitude, subjective norm, and perceived behavioral control of khat chewing. Behavioral intention to khat chewing was primarily under the attitudinal influence. The indirect measures of TPB had influences on direct measures of TPB, so that intention to khat chewing will be increased. Increasing health literacy by transferring health messages in media outlets and giving particular emphasis on risk perceptions of khat chewing. A prospective study design is recommended to determine the relationships between constructs; measuring behavior at time intervals as human behavior cannot be always stable. It is better if construct validity is done for the data collection tool. Large-scale qualitative study is suggested by involving religious leaders, community leaders, farmers, agriculture personnel, and health professionals.

## Data Availability

The datasets presented in this study can be found in online repositories. The names of the repository/repositories and accession number(s) can be found in the article/supplementary material.

## References

[1] Megerssa B Esayas A Mohamed A. Socio-economic impact of Khat in Mana District, Jimma zone, South Western Ethiopia. Agric Sci. (2013) 1:44–59.

[2] Lim SYM Alshagga MA Ong CE Pan Y. Naturally occurring cathinone from Khat, synthetic Cathinones and cytochrome P450 In: Patel VB Preedy VR, editors. Handbook of substance misuse and addictions: from biology to public health. Cham: Springer International Publishing (2021). 1–23.

[3] Lim SYM Alshagga MA Alshawsh MA Ong CE Pan Y. In vitro effects of 95% khat ethanol extract (KEE) on human recombinant cytochrome P450 (CYP)1A2, CYP2A6, CYP2B6, CYP2C8, CYP2C19, CYP2E1, CYP2J2 and CYP3A5. Drug Metab Pers Ther. (2022) 37:55–67. doi: 10.1515/dmpt-2021-1000196, PMID: 35146975

[4] Wondemagegn AT Cheme MC Kibret KT. Perceived psychological, economic, and social impact of Khat chewing among adolescents and adults in Nekemte town, east Welega zone, West Ethiopia. Biomed Res Int. (2017) 2017:1–9. doi: 10.1155/2017/7427892, PMID: 28265577 PMC5317140

[5] Gebrie A Alebel A Zegeye A Tesfaye B. Prevalence and predictors of khat chewing among Ethiopian university students: a systematic review and meta-analysis. PLoS One. (2018) 13:e0195718. doi: 10.1371/journal.pone.0195718, PMID: 29649253 PMC5896981

[6] Al-Juhaishi T Al-Kindi S Gehani A. Khat: a widely used drug of abuse in the horn of Africa and the Arabian peninsula: review of literature. Qatar Med J. (2013) 2012:1–6. doi: 10.5339/qmj.2012.2.5, PMID: 25003033 PMC3991038

[7] ECDD. Assessment of khat (*Catha edulis* Forsk). Geneva: WHO (2006).

[8] Ware E Tura G Alemu T Andarge E. Disparities in risky sexual behavior among khat chewer and non- chewer college students in southern Ethiopia: a comparative cross-sectional study. BMC Public Health. (2018) 18:558. doi: 10.1186/s12889-018-5405-x, PMID: 29703181 PMC5921970

[9] Central Statistical Agency and ICF. Ethiopia demographic and health survey 2016. Rockville, Maryland: Central Statistical Agency and ICF; (2017). 1–551.

[10] Fekadu A Alem A Hanlon C. Alcohol and drug abuse in Ethiopia: past, present and future. Afr J Drug Alcohol Stud. (2007) 6:40–53.

[11] Ethiopian Public Health Association. Legal aspects of substance and alcohol abuse in Ethiopia, Ethiopia: Ethiopian Public Health Association; (2011) 1–17.

[12] Prochaska JO Velicer WF. The Transtheoretical model of health behavior change. Am J Health Promot. (1997) 12:38–48. doi: 10.4278/0890-1171-12.1.38, PMID: 10170434

[13] Fidancı İ Ozturk O Unal M. Transtheoretic model in smoking cessation. J Exp Clin Med Turk. (2017) 34:9–13. doi: 10.5835/jecm.omu.34.01.003

[14] Girma E Assefa T Deribew A. Cigarette smokers’ intention to quit smoking in Dire Dawa town Ethiopia: an assessment using the Transtheoretical model. BMC Public Health. (2010) 10:320. doi: 10.1186/1471-2458-10-320, PMID: 20529337 PMC2889871

[15] Adugna A Azale T Handebo S. Seven in every ten khat chewers in Gondar City had an intention to stop khat chewing: cross-sectional study using Transtheoretical model. BMC Psychiatry. (2020) 20:577. doi: 10.1186/s12888-020-02984-4, PMID: 33267853 PMC7709406

[16] Estifanos M Azale T Slassie M Aynalem G Kefale B. Intention to stop Khat chewing and associated factors among Khat chewers in Dessie City, north eastern Ethiopia. Epidemiol Open Access. (2016) 6:11–6. doi: 10.4172/2161-1165.1000250, PMID: 37784109

[17] Winfree LT Griffiths CT Sellers CS. Social learning theory, drug use, and American Indian youths: a cross-cultural test. Justice Q. (1989) 6:395–417. doi: 10.1080/07418828900090271

[18] Durkin KF Wolfe TW Clark GA. College students and binge drinking: an evaluation of social learning theory. Sociol Spectr. (2005) 25:255–72. doi: 10.1080/027321790518681

[19] Akers RL Lee G. Age, social learning, and social bonding in adolescent substance use. Deviant Behav. (1999) 20:1–25. doi: 10.1080/016396299266579

[20] Simons RL Conger RD Whitbeck LB. A multistage social learning model of the influences of family and peers upon adolescent substance abuse. J Drug Issues. (1988) 18:293–315. doi: 10.1177/002204268801800301

[21] Akers RL Lee G. A longitudinal test of social learning theory: adolescent smoking. J Drug Issues. (1996) 26:317–43. doi: 10.1177/002204269602600203

[22] Ajzen I. From intentions to actions: a theory of planned behavior In: Kuhl J Beckmann J, editors. Action control: from cognition to behavior. Berlin, Heidelberg: Springer Berlin Heidelberg (1985). 11–39.

[23] Norberg PA Horne DR Horne DA. The privacy paradox: personal information disclosure intentions versus Behaviors. J Consum Aff. (2007) 41:100–26. doi: 10.1111/j.1745-6606.2006.00070.x

[24] Ajzen I. The theory of planned behavior. Organ Behav Hum Decis Process. (1991) 50:179–211. doi: 10.1016/0749-5978(91)90020-T

[25] Rise J Sheeran P Hukkelberg S. The role of self-identity in the theory of planned behavior: a meta-analysis. J Appl Soc Psychol. (2010) 40:1085–105. doi: 10.1111/j.1559-1816.2010.00611.x

[26] Francis JJ Eccles MP Johnston M Walker A Grimshaw J Foy R. Constructing questionnaires based on the theory of planned behaviour: A manual for health services researchers. Newcastle upon Tyne: Centre for Health Services Research, University of Newcastle (2004).

[27] Abdeta T Tolessa D Adorjan K Abera M. Prevalence, withdrawal symptoms and associated factors of khat chewing among students at Jimma University in Ethiopia. BMC Psychiatry. (2017) 17:142. doi: 10.1186/s12888-017-1284-4, PMID: 28412950 PMC5392995

[28] Cooke R Dahdah M Norman P French DP. How well does the theory of planned behaviour predict alcohol consumption? A systematic review and meta-analysis. Health Psychol Rev. (2016) 10:148–67. doi: 10.1080/17437199.2014.947547, PMID: 25089611 PMC4867851

[29] Topa G Moriano JA. Theory of planned behavior and smoking: meta-analysis and SEM model. Subst Abus Rehabil. (2010) 1:23–33. doi: 10.2147/SAR.S15168, PMID: 24474850 PMC3819188

[30] Ganley BJ Rosario DI. The smoking attitudes, knowledge, intent, and behaviors of adolescents and young adults: implications for nursing practice. J Nurs Educ Pract. (2013) 3:40–50. doi: 10.5430/jnep.v3n1p40

[31] Huchting K Lac A LaBrie JW. An application of the theory of planned behavior to sorority alcohol consumption. Addict Behav. (2008) 33:538–51. doi: 10.1016/j.addbeh.2007.11.002, PMID: 18055130 PMC2387076

[32] Alanazi NH Lee JW Dos Santos H Job JS Bahjri K. The use of planned behavior theory in predicting cigarette smoking among Waterpipe smokers. Tob Induc Dis. (2017) 15:29. doi: 10.1186/s12971-017-0133-z, PMID: 28690480 PMC5496426

[33] Glanz K Rimer BK Viswanath K Orleans CT. Health behavior and health education: Theory, research, and practice. San Francisco, CA: Jossey-Bass (2008).

[34] Kandari LS Yadav HR Thakur AK Kandari T. Khat (*Catha edulis*): a socio economic crop in Harar region, eastern Ethiopia. Springerplus. (2014) 3:579. doi: 10.1186/2193-1801-3-579, PMID: 25332879 PMC4197201

[35] Mihretu A Teferra S Fekadu A. What constitutes problematic khat use? An exploratory mixed methods study in Ethiopia. Subst Abuse Treat Prev Policy. (2017) 12:17. doi: 10.1186/s13011-017-0100-y, PMID: 28327160 PMC5361726

[36] Yahya A Rajeshwar Y Eticha T Kahsay G Ali D Gebretsadik H. Socio-economic and health effects of Khat chewing in Mekelle, Tigray region, Ethiopia. Int J Pharm Pharm Res. (2016) 8:11–22.

[37] Wiedyaningsih C Hakimi M Soenarto Y Suryawati S. The use of the theory of planned behavior to predict factors influencing physicians’ decision to prescribe extemporaneous compounding dosage form for pediatric outpatients. Asian J Pharm Clin Res. (2016) 9:288–91.

[38] Lee J Cerreto F A Lee J. Theory of planned behavior and teachers’ decisions regarding use of educational technology. Educ Technol Soc. (2010) 13:152–64.

